# Phylogenomic, Pan-genomic, Pathogenomic and Evolutionary Genomic Insights into the Agronomically Relevant Enterobacteria *Pantoea ananatis* and *Pantoea stewartii*

**DOI:** 10.3389/fmicb.2017.01755

**Published:** 2017-09-14

**Authors:** Pieter De Maayer, Habibu Aliyu, Surendra Vikram, Jochen Blom, Brion Duffy, Don A. Cowan, Theo H. M. Smits, Stephanus N. Venter, Teresa A. Coutinho

**Affiliations:** ^1^School of Molecular and Cell Biology, University of the Witwatersrand Johannesburg, South Africa; ^2^Centre for Microbial Ecology and Genomics, Genomics Research Institute, University of Pretoria Pretoria, South Africa; ^3^Department of Bioinformatics and Systems Biology, Justus-Liebig-University Giessen Giessen, Germany; ^4^Environmental Genomics and Systems Biology Research Group, Institute for Natural Resource Sciences, Zurich University of Applied Sciences Winterthur, Switzerland; ^5^Department of Microbiology, Forestry and Agricultural Biotechnology Institute, University of Pretoria Pretoria, South Africa

**Keywords:** Stewart’s wilt, mobilome, pan-genome, phylogenomics, pathogenicity factors, host range, pathoadaptation

## Abstract

*Pantoea ananatis* is ubiquitously found in the environment and causes disease on a wide range of plant hosts. By contrast, its sister species, *Pantoea stewartii* subsp. *stewartii* is the host-specific causative agent of the devastating maize disease Stewart’s wilt. This pathogen has a restricted lifecycle, overwintering in an insect vector before being introduced into susceptible maize cultivars, causing disease and returning to overwinter in its vector. The other subspecies of *P. stewartii* subsp. *indologenes*, has been isolated from different plant hosts and is predicted to proliferate in different environmental niches. Here we have, by the use of comparative genomics and a comprehensive suite of bioinformatic tools, analyzed the genomes of ten *P. stewartii* and nineteen *P. ananatis* strains. Our phylogenomic analyses have revealed that there are two distinct clades within *P. ananatis* while far less phylogenetic diversity was observed among the *P. stewartii* subspecies. Pan-genome analyses revealed a large core genome comprising of 3,571 protein coding sequences is shared among the twenty-nine compared strains. Furthermore, we showed that an extensive accessory genome made up largely by a mobilome of plasmids, integrated prophages, integrative and conjugative elements and insertion elements has resulted in extensive diversification of *P. stewartii* and *P. ananatis*. While these organisms share many pathogenicity determinants, our comparative genomic analyses show that they differ in terms of the secretion systems they encode. The genomic differences identified in this study have allowed us to postulate on the divergent evolutionary histories of the analyzed *P. ananatis* and *P. stewartii* strains and on the molecular basis underlying their ecological success and host range.

## Introduction

The enterobacterial genus *Pantoea* currently encompasses twenty-two distinct species^1^. A key feature of this genus is the extensive ecological and functional diversity of its members, testament to the Greek derivation of the genus name – Gr. adj. *pantoios*, of all sorts and sources (Gavini et al., 1989; Mergaert et al., 1993; Walterson and Stavranides, 2015). *Pantoea* strains have been isolated globally from diverse ecological sources, both natural and man-made, and have diverse lifestyles, including as commensals, insect symbionts and as plant and clinical pathogens (Mergaert et al., 1993; Walterson and Stavranides, 2015). There has also been increased interest in *Pantoea* strains from a biotechnological perspective, including in bioremediation strategies, the production of novel medical therapeutics and as biological control agent against insect pests and plant pathogens (Walterson and Stavranides, 2015; Weller-Stuart et al., 2016).

The species *Pantoea ananatis* (*Pnan*) exemplifies the ecological and functional diversity of the genus, as strains have been isolated from a wide range of environmental sources, including water, soil and man-made environments and have diverse lifestyles, including as insect commensals, clinical pathogen and as part of the epiphytic and endophytic communities on a very broad range of plant hosts (Coutinho and Venter, 2009; Weller-Stuart et al., 2016). Comparative genomic analyses have revealed a wide range of factors underlying their ecological success and ability to interact with different hosts (Weller-Stuart et al., 2016). Individual strains of *Pnan* have also been shown to have plant growth promoting capabilities, anti-bacterial and anti-fungal properties, and bioremediation and biofuel production capacities and as such there has been much recent interest in the biotechnological application of this species (Walterson and Stavranides, 2015; Weller-Stuart et al., 2016). *Pnan* is, however, best known as an emerging, and potentially opportunistic, pathogen on an extremely wide spectrum of plant hosts, including rice, maize, onions and *Eucalyptus* where it causes an equally vast array of disease symptoms, including stalk, fruit and bulb rot, leaf spot, blight and dieback (Coutinho and Venter, 2009; Weller-Stuart et al., 2016). To date, relatively little is known about the mechanisms of pathogenesis, epidemiology and spread, although insects such as tobacco thrips and cotton fleahoppers have been described as potential vectors of *Pnan* strains (Coutinho and Venter, 2009).

By contrast to *Pnan*, the closely related species *P. stewartii* (*Pnst*) incorporates the aggressive phytopathogen *Pantoea stewartii* subsp. *stewartii* which is host specific to maize (*Zea mays*), in particular sweet corn (*Z. mays* convar. *saccharata* var. *rugosa*) and inbred lines of maize (Roper, 2011). Stewart’s wilt of maize is a devastating disease that results in severe crop losses of susceptible varieties and is endemic to the United States, and as a result quarantine restrictions are in place to prevent spread of this pathogen (Roper, 2011). Given the agronomic importance of *Pnst* subsp. *stewartii*, its epidemiology, pathogenesis and means of disease control have been extensively researched. By contrast to *Pnan, Pnst* subsp. *stewartii* has a restricted lifecycle, overwintering in the corn flea beetle (*Chaetocnema pulicularia*), which subsequently introduces the pathogen into feeding wounds via its feces when it emerges in spring (Roper, 2011; Correa et al., 2012). *Pnst* subsp. *stewartii* subsequently colonizes the interstitial spaces in maize leaf tissues, where it causes the development of water-soaked lesions, and subsequently the xylem, resulting in systemic spread and wilting. The pathogen is then acquired by the last seasonal generation of the corn flea beetle in which it again overwinters (Roper, 2011). The other member of this species, *Pantoea stewartii* subsp. *indologenes*, is avirulent on maize and was long considered as non-pathogenic, although it has been linked to symptom development on pearl and foxtail millet, *Eucalyptus*, cluster bean and rot of pineapple fruit, suggesting that unlike the *Pnst* subsp. *stewartii* this subspecies has a broader host range (Mergaert et al., 1993; Coutinho et al., 2002). As with *Pnan*, little is known about the epidemiology and pathogenicity determinants of *Pnst* subsp. *indologenes*. Furthermore, two *Pnst* strains, which have not been characterized to the subspecies level, were isolated from a waterfall (Izzati Mohamad et al., 2015; Tan et al., 2015), suggesting that at least some isolates of this species are capable of surviving in the environment outside of a plant or insect host.

The distinct lifestyles and ecological versatility of *Pnan* and the *Pnst* subspecies raises several questions about the evolution of these bacteria. How has *Pnst* subsp. *stewartii* evolved into a host-specific pathogen on maize, while *Pnan* and *Pnst* subsp. *indologenes* cause disease on a broad range of plant hosts? Why is the lifecycle of *Pnst* subsp. *stewartii* largely restricted to its maize and insect hosts, while *Pnan* and other *Pnst* strains are frequently isolated from a wide range of natural sources and plant hosts? Do these pathogens make use of different pathogenicity factors for symptoms development? With the advent of cost-effective genome sequencing, comparative genomic approaches provide a viable approach to answering these questions. For example, host-specificity of *Pseudomonas syringae* strains isolated from different plant hosts was correlated, in part, to differences in their T3SS effector and phytotoxin production profiles by genome comparisons and genetic manipulation (Baltrus et al., 2001, 2012). Similar studies incorporating population genetics and comparative genomics have been undertaken to study genetic variation between host-adapted and broad host range *Ralstonia solanacearum* strains (Ailloud et al., 2015), and the distinct ecologies, physiologies and host specificities among *Xanthomonas* strains (Jacques et al., 2016). A previous pan-genome analysis of eight *Pnan* strains also revealed distinct factors which may influence their ability to interact with plant, insect, and animal hosts (De Maayer et al., 2014). Here we have employed comparative genomic analyses and a broad array of *in silico* tools to compare the genomes of nineteen *Pnan* and ten *Pnst* strains. Our analyses revealed a range of different evolutionary drivers, as well as strain- and species-specific factors which may explain the distinct lifestyles of *Pnan* and *Pnst* strains, and expand our understanding of these two important agronomic species.

## Materials and Methods

### Genomic Analyses

The genome sequences of nineteen *Pnan* and ten *Pnst* strains were obtained from the NCBI genome database^2^ (**Table 1**). The genome assemblies were refined by genome alignment using MAUVE v. 2.4.0 (Darling et al., 2010) and genome scaffolding using the MeDuSa web server (Bosi et al., 2015). General sequence manipulations, G+C % calculations and local BlastP searches were undertaken using BioEdit v. 7.2.5 (Hall, 1999). The complete and high quality draft genomes were submitted to the RAST v 2.0 server (Aziz et al., 2008) for structural and functional annotation. The genome annotations and local BlastP searches were used to identify putative plasmid (BlastP analyses with Rep and Mob proteins) sequences, ICE elements, transposases and pathogenicity determinants. For the BlastP analyses, orthologous proteins were identified as those sharing >50% amino acid identity and alignment coverage of >70%. Transposases were further categorized at the family level by identifying the best Blast hits from comparison against the Conserved Domain Database using CD-Search (Marchler-Bauer and Bryant, 2004). Similarly, integrated prophages were identified using the PHAST server (Zhou et al., 2011). Insertion Sequences were identified and analyzed using the ISSaga server (Varani et al., 2011).

**Table 1:** General genome properties of the twenty-nine compared *Pnan* and *Pnst* strains.

Species	Strain	Source	Geographic	Genome	# Contigs	Size (Mb)	G+C	# CDSs	Reference	
			location	NCBI Acc. #			content (%)			
*Pnan1*	AJ13355	Soil	Japan	NC_017531/3	2	4.88	53.64	4671	Hara et al., 2012	
	Bl-9	Onion	Korea	CAEI01000000	15	5.11	53.48	4951	Kim et al., 2012	
	BD442	Maize	South Africa	JMJL01000000	11	4.8	53.59	4645	Weller-Stuart et al., 2014	
	BRT175	Strawberry	Canada	ASJH01000000	12	4.83	53.74	4649	Smith et al., 2013	
	DAR 76143	Rice	Australia	BATH01000000	28	5.22	53.43	5778	–	
	LMG 2665^T^	Pineapple	United States	JFZU01000000	9	4.8	53.39	4782	Adam et al., 2014	
	LMG 5342	Human	Phillipines	NC_016816/7	2	4.91	53.32	4715	De Maayer et al., 2012b	
	LMG 20103	*Eucalyptus*	South Africa	NC_013956	2	4.7	53.69	4475	De Maayer et al., 2010	
	NFR11	Switchgrass	United States	2599185299^a^	10	4.86	53.47	4714	–	
	PA4	Onion	South Africa	JMJK01000000	16	5.15	53.55	5070	Weller-Stuart et al., 2014	
	PA13	Rice	Korea	NC_017553/4	2	4.87	53.58	4709	Choi et al., 2012	
	R100	Rice seed	China	NC_CP014207/8	2	4.86	53.62	4656	Wu et al., 2016	
	S7	Maize seed	Austria	CVNG01000000	11	5.03	53.51	4897	Sheibani-Tezerji et al., 2015	
	S8	Maize seed	Austria	CVNH01000000	13	4.94	53.56	4760	Sheibani-Tezerji et al., 2015	
*Pnan2*	NS296	Rice seed	India	LDQX01000000	16	4.74	53.53	4650	Midha et al., 2016	
	NS303	Rice seed	India	LDQY01000000	16	4.74	53.53	4648	Midha et al., 2016	
	NS311	Rice seed	India	LDQZ01000000	16	4.74	53.53	4654	Midha et al., 2016	
	RSA47	Rice seed	India	LDRA01000000	17	4.74	53.53	4651	Midha et al., 2016	
	Sd-1	Rice seed	China	AZTE01000000	22	4.95	53.34	4838	Shi et al., 2015	
*Pnst*	A206	Peach palm	Costa Rica	LIHC01000000	12	4.59	53.82	4421	–	
	DC283	Maize	United States	AHIE01000000	53	5.22	53.83	5487	–	
	LMG 2632^T^	Foxtail millet	India	JPKO01000000	22	4.68	53.70	4642	–	
	M009	Waterfall	Malaysia	JRWI01000000	12	4.82	53.90	4700	Tan et al., 2015	
	M073a	Waterfall	Malaysia	JSFX01000000	9	4.82	53.91	4693	Izzati Mohamad et al., 2015	
	NS381	Rice seed	India	LDSH01000000	16	4.69	53.87	4534	Midha et al., 2016	
	RSA13	Rice seed	India	LDSI01000000	11	4.77	53.68	4603	Midha et al., 2016	
	RSA30	Rice seed	India	LDSJ01000000	11	4.76	53.68	4614	Midha et al., 2016	
	RSA36	Rice seed	India	LDSK01000000	11	4.77	53.68	4606	Midha et al., 2016	
	S301	Peach palm	Costa Rica	LIIU01000000	12	4.48	53.93	4307	–	

*The number of contigs reflects those as assembled in this study. ^a^Denotes the genome accession number as per the Integrated Microbial Genome Database project (IMG ID: 2599185299) from which the data was derived.*

### Comparative Genomic Analyses

The annotated GenBank files from RAST were uploaded to the EDGAR 2.1 comparative genomic pipeline (Blom et al., 2016). Orthologous proteins among the compared genomes were identified on the basis of BLAST Score Ratio Values (SRVs) as implemented in EDGAR (Blom et al., 2009). Core proteins (shared among individual *Pnan1*+ *Pnan2* + *Pnst* strains and among all twenty-nine compared strains), proteins shared among strains belonging to two groups (*Pnan1* + *Pnan2*; *Pnan1* + *Pnst*; *Pnan2* + *Pnst*) and unique proteins (unique to *Pnan1, Pnan2* or *Pnst* strains) were identified. The proteins in each of these datasets were further assigned to their Conserved Orthologous Groups supra-functional (Cellular processes; Information storage and processing; metabolism; poorly characterized) and functional categories on the basis of the HMM models for Bacteria (bactNOG 4.5) using hmmscan (Huerta-Cepas et al., 2016). Orthologous pathogenicity determinants identified from the annotated genomes were identified in EDGAR 2.1 (Blom et al., 2016) and average amino acid identity values determined using local BlastP analyses in BioEdit v 7.2.5 (Hall, 1999). Orthology was assumed for those proteins sharing >50% amino acid identity and alignment coverage of >70%.

### Phylogenomic Analyses

A core genome phylogeny was constructed via the EDGAR 2.1 pipeline (Blom et al., 2016), on the basis of 2,817 core genes conserved among the compared *Pnan1, Pnan2* and *Pnst* genomes as well as the outgroup strain *P. vagans* C9-1 (Smits et al., 2011). Core genes were aligned using MUSCLE (Edgar, 2004), non-matching regions in the alignments were removed using GBLOCKS (Talavera and Castresana, 2007), and the aligned sequences were concatenated and used to generate a neighbor-joining tree with Phylip (Felsenstein, 1989). The phylogeny was visualized using MEGA v 7.0.14 (Kumar et al., 2016). The two-way ANI values were calculated for the core genes for each pair of compared strains using the ani.rb script included in the Enveomics package (Rodriguez-R and Konstantinidis, 2016). Digital DNA-DNA hybridization (dDDH) values were estimated using the Genome-to-Genome Distance Calculator 2.0^3^, with the recommended formula 2 (Auch et al., 2010; Meier-Kolthoff et al., 2013).

## Results

### *Pantoea ananatis* and *Pantoea stewartii* Genome Properties

The complete or high quality draft genomes of ten *Pnst* and nineteen *Pnan* strains were included in this study (**Table 1**). The draft genomes were assembled to between nine (Pnan LMG 2665^T^ and *Pnst* M073a) and fifty-three contigs (*Pnst* DC283). The *Pnst* and *Pnan* genomes have an average size of 4.89 and 4.76 Mb, respectively. The genomes of three *Pnan* strains are substantially larger than the other sixteen compared strains (B1-9: 5.11 Mb; PA4: 5.15 Mb; and DAR76143: 5.23 Mb). Similarly, the draft genome of *Pnst* DC283 (5.23 Mb) is substantially larger than those of the other nine *Pnst* strains (average size: 4.71 Mb). Concomitantly, an additional ∼918 protein coding sequences (CDSs) are present on the *Pnst* DC283 genome (5,487 CDSs) compared to the other nine *Pnst* strains (average 4,568 CDSs). The highest number of proteins (5,778 CDSs) are encoded on the genome of *Pnan* DAR76143, while the strain with the smallest genome, *Pnst* S301 (4.48 Mb), encodes 4,307 proteins (**Table 1**). Slightly higher G+C contents were observed for the genomes of the *Pnst* strains (average G+C content: 53.80%) than those of the *Pnan* strains (average G+C content: 53.53%).

### Phylogenomic Analyses Reveal the Existence of Two Distinct *P. ananatis* Clades

**FIGURE 1:**
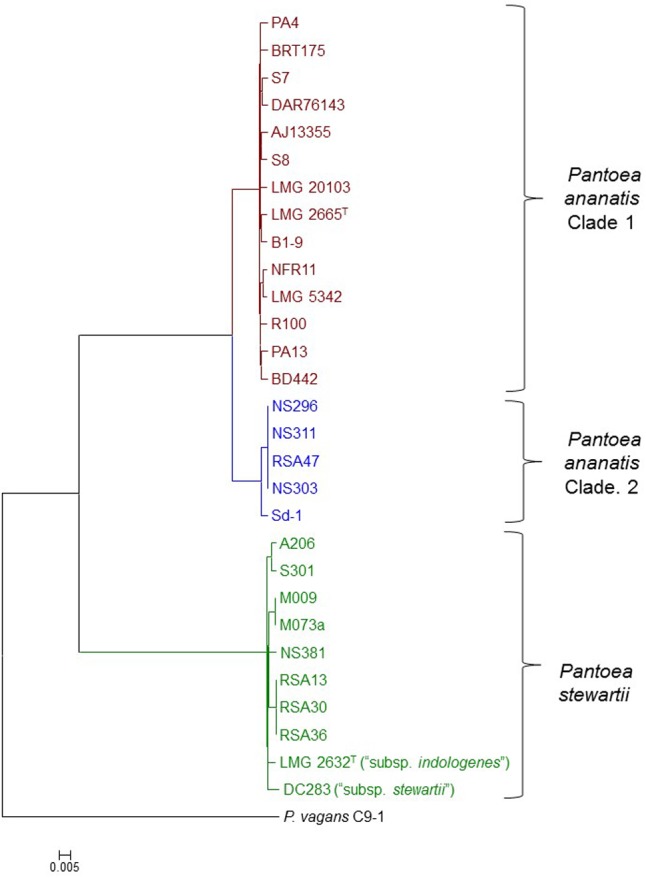
Core genome phylogeny of twenty-nine compared *Pnan* and *Pnst* strains. The core genome phylogeny was constructed on the basis of 2,817 core genes conserved among all compared strains via the EDGAR 2.1 pipeline (Blom et al., 2016). The genome of *P. vagans* C9-1 (Smits et al., 2011) (NCBI Acc. # CP002206.1; CP001893/4/5.1) was used as outgroup.

A core genome phylogeny was constructed on the basis of 2,810 genes conserved in all *Pnan* and *Pnst* strains used in this study, as well as the outgroup strain *P. vagans* C9-1 (Smits et al., 2011). This phylogeny showed distinct clustering of the *Pnan* and *Pnst* strains. Two distinct clades could be observed among the *Pnan* strains, with the first clade encompassing fourteen strains, including the type strain Pnan LMG 2665^T^, while the second clade incorporates five strains (**Figure 1**). dDDH values were determined using the Genome-to-Genome Distance Calculator (GGDC) tool (**Supplementary Table S1**) (Auch et al., 2010). On the basis of parameters for species delineation deemed valid for both wet-lab DNA-DNA hybridization and dDDH (organisms displaying DDH values < 70% are considered as distinct species) (Meier-Kolthoff et al., 2013), the nineteen *Pnan* strains and ten *Pnst* strains can be considered as distinct species (average dDDH values of 27.77 ± 0.20%; dDDH ± standard deviation). ANI threshold values of 95–96% have also been used to demarcate distinct species (Goris et al., 2007) and the average ANI value of 84.26 ± 0.14% (Average ANI value ± standard deviation) observed in this study (**Supplementary Table S1**) further support the delineation of *Pnan* and *Pnst* as distinct species.

The five *Pnan* strains in the second clade of the core genome phylogeny share an average dDDH value of 69.89 ± 1.11% and average ANI value of 96.32 ± 0.10 % with the fourteen strains in the other *Pnan* clade (**Figure 1**), while the latter strains share average dDDH and ANI values of 92.04 ± 1.81% and 99.10 ± 0.13%, respectively (**Supplementary Table S1**). The dDDH values suggests that the strains in the second clade are on the borderline for forming a distinct species, while the lower ANI values provide support that strains in this clade are genetically distinct from strains in the other *Pnan* clade. Further phenotypic and biochemical characterization will need to be undertaken to validate the phylogenomic observations and to describe the potential novel species, as metadata for many of the strains are lacking. For the purpose of further comparative genomic, mobilomic and pathogenomic characterization of the *Pnan* and *Pnst* strains, we have distinguished the five *Pnan* strains (NS296, NS303, NS311, RSA47, and Sd-1) in the second clade as *P. ananatis* Clade 2 (*Pnan2*) while the remaining fourteen *P. ananatis* strains form part of *P. ananatis* Clade 1 (*Pnan1*).

Far higher average ANI (99.14 ± 0.29%) and dDDH (92.76 ± 2.53%) values were observed when comparing the ten *Pnst* strains (**Supplementary Table S1**), suggesting that less phylogenetic diversity exists between within this species and raising questions about their delineation as distinct subspecies (Meier-Kolthoff et al., 2014). This is, however, in contrast to the wet-lab DDH values observed (79%) when the genomic DNA of *Pnst* subsp. *indologenes* LMG 2632^T^ was compared with that of the type strain of *Pnst* subsp. *stewartii* LMG 2715^T^ and clear phenotypic differences were observed between these two subspecies in the original species description (Mergaert et al., 1993). We have therefore maintained the original subspecies nomenclature for the further comparative genomic analyses while the remaining eight *Pnst* strains were not delineated at the subspecies level.

### Pan-genome Analyses Reveal Extensive Interspecies and Inter-subspecies Variability and Functional Diversity among *Pnan* Clade 1, *Pnan* Clade 2 and *Pnst*

**FIGURE 2:**
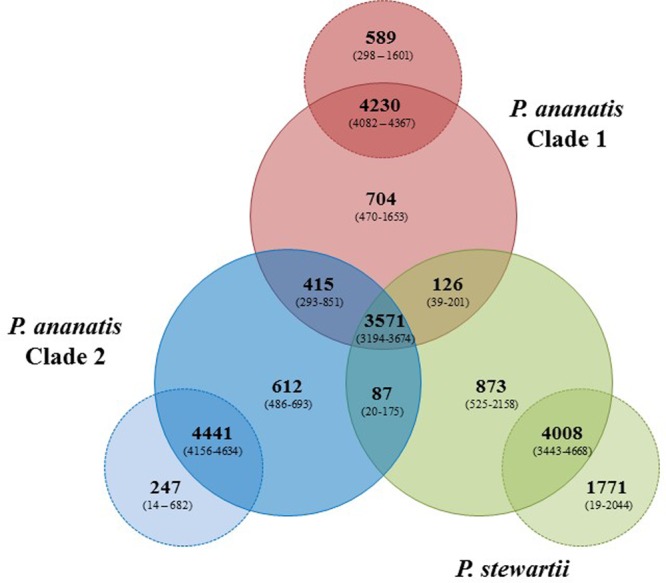
Pan-genome Venn diagram of the twenty-nine compared *Pnan* and *Pnst* strains. The average number (bold large) and the range (in brackets) of proteins comprising the core (in *Pnan1*+*Pnan2*+*Pnst* strains), accessory shared (between *Pnan1*+*Pnan2*; *Pnan1*+*Pnst*; *Pnan2*+*Pnst*) and accessory unique genome fractions of the three-ways compared *Pnan1, Pnan2* and *Pnst* strains, as well as the average and range of proteins that are core and accessory within the three distinct groups are shown.

Pan-genome analyses were undertaken using the EDGAR 2.1 pipeline, where orthologs were identified on the basis of Blast SRV (Blom et al., 2009). Individual *Pnan1, Pnan2* and *Pnst* strains were three-way compared and average values for the core and accessory genome fractions were determined (**Figure 2**). An extensive core genome, consisting of an average of 3,571 CDSs (range 3,194–3,674 CDSs) was observed for the three-way comparison of the twenty-nine strains. This constitutes between 55.28 and 83.93% of the CDSs encoded on each genome, with the lower values reflecting those strains with larger genomes, including *Pnan1* strains B1-9 and DAR76143 and *Pnst* DC283, while the higher value reflects the smallest genome of *Pnst* S301. It is notable that the core genome is considerably smaller when considering *Pnan1*+ *Pnan2* + *Pnst* DC283 (average core CDSs = 3,241) than *Pnan1*+ *Pnan2* + all other *Pnst* strains (average core CDSs = 3,612 CDSs).

Analysis of the shared accessory fractions showed a substantial number of proteins shared only between the *Pnan1* and *Pnan2* strains (average: 415 CDSs; range 293–851 CDSs), which may be correlated to their closer phylogenetic relationship. By contrast, few proteins are shared between *Pnan1*+ *Pnst* and *Pnan2*+ *Pnst* strains, respectively. Similar proportions of proteins are unique to the *Pnan1, Pnan2*, and *Pnst* groups (average unique CDSs = 704, 612, and 873 CDSs, respectively) (**Figure 2**). Pair-wise comparisons between strains in each of the three groups were also undertaken. While large core genomes were observed for combinations of strains in the *Pnan1, Pnan2* and *Pnst* clusters, lower intraspecific variability (average unique CDSs = 247; range 14–682 CDSs) exists among *Pnan2* strains. This may be linked to the fact that four of the five *Pnan2* strains analyzed were isolated from rice seeds in the same study (Midha et al., 2016). Many more unique CDSs could be observed when comparing *Pnan1* strains (average unique CDSs = 589; range 298–1601 CDSs). The highest number of unique CDSs was, however, observed among the *Pnst* strains (average unique CDSs = 1,771 CDSs; range 525–2158 CDSs) (**Figure 2**). This could mainly be attributed to the maize pathogenic strain *Pnst* DC283. Comparisons of *Pnst* DC283 and other *Pnst* strains showed that between 1,956 and 2,044 *Pnst* DC283 CDSs have no orthologs in the other *Pnst* strains. This highlights the extensive intra-specific variability, in terms of protein content, among strains of *P. ananatis* and *P. stewartii*, particularly among the *Pnan1* and *Pnst* strains, while the lower intra-specific variability in the *Pnan2* clade may reflect clonality among four of the five studied strains that were isolated from rice seeds.

**FIGURE 3:**
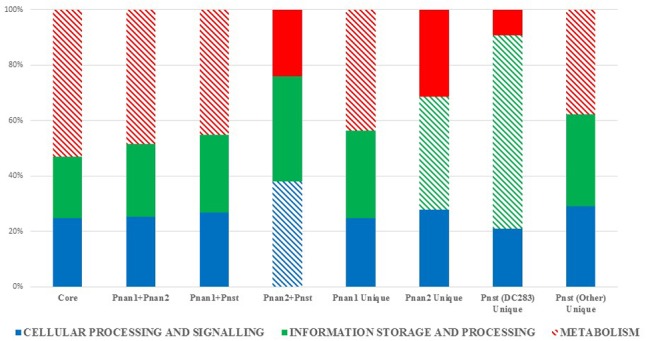
Super-functional classification of the core, accessory shared and unique proteins among the compared *Pnan1, Pnan2* and *Pnst* strains. The relative percentages of proteins involved in the super-functional categories Cellular processing and signaling (blue), Information processing and storage (green) and Metabolism (red) were calculated. The predominant fraction is indicated with cross-hatched filling.

The protein datasets for the core and accessory fractions were extracted and subjected to local HMMER search against the eggNOG 4.5 BactNOG database (Huerta-Cepas et al., 2016). By this means the proteins in the distinct datasets were classified according to their Conserved Orthologous Groups functional and super-functional categories (Tatusov et al., 2000; De Maayer et al., 2014) and the relative proportions of proteins involved in each distinct functional category were determined (**Figure 3**). Proteins belonging to the “poorly characterized” super-functional category were removed as these make up the majority of proteins in each dataset and thus dilute the overall differences in the other super-functional categories. The core genome is predominated by proteins in the “metabolism” super-functional category. Similarly, the accessory fractions shared by *Pnan1*+ *Pnan2* (but not *Pnst*) strains and *Pnan1*+ *Pnst* (but not *Pnan2*) are also dominated by proteins involved in metabolism. By contrast, more proteins in the “cellular processing and signaling” super-functional category are shared by the *Pnan2*+ *Pnst* strains. The proteins unique to the *Pnan1* strains and *Pnan2* strains are mainly involved in metabolism and information processing and storage, respectively (**Figure 3**). This difference is mainly due to the much higher number of proteins involved in metabolism in *Pnan1* strains, in particular nucleotide transport and metabolism (3.2× as many proteins in *Pnan1* than in the others) and lipid transport and metabolism (3.1× as many proteins in *Pnan1* than in the others), while relatively similar numbers of proteins are involved in information storage and processing. One key observation from this analysis was the difference between the accessory fraction of *Pnst* DC283 and that of the other *Pnst* strains (**Figure 3**). While relatively similar numbers of proteins are involved in metabolism, much higher numbers of proteins are involved in information processing and signaling in *Pnst* DC283 than in the other *Pnst* strains. This can largely be attributed to those proteins involved in DNA replication, recombination and repair, with an average of nineteen proteins involved in this function in the other *Pnst* strains, while an average of 398 proteins fall in this category in *Pnst* DC283. This will be discussed in more detail below.

### Plasmid Maintenance, Phage and ICE Element Integration and Transposable Elements Are Key Drivers of Evolutionary Diversification

#### Plasmids

A particular feature of *Pnst* subsp. *stewartii* strains is their ability to maintain large numbers of plasmids, with between eleven and thirteen plasmids, ranging in size from 4 to 320 kb, have previously been identified in 39 virulent strains by gel electrophoresis (Coplin et al., 1981). It was postulated that up to 25% of the total *Pnst* subsp. *stewartii* genomic DNA content may be plasmid-borne (Coplin et al., 1981). Using available sequences for *Pnst* subsp. *stewartii* plasmids, as well as searching for proteins involved in plasmid replication and maintenance, potential plasmids were identified in *Pnst* DC283. By this means, copies of four previously characterized *Pnst* plasmids, pSW100 (AHIE01000052.1; 4.3 kb; circular), pSW200 (AHIE01000062.1; 4.4 kb; circular), pSW800 (AHIE01000065.1; 34.4 kb; circular) and pSW1200 (AHIE01000039.1; 115.4 kb; incomplete) were identified (Fu et al., 1995, 1997, 1998; Wu et al., 2001). One further complete circular plasmid (AHIE01000057.1; 13.4 kb) was found. In addition, five further contigs containing either *rep, tra*/*trb* or *mob* genes were identified. We have previously analyzed a plasmid, LPP-1 (Large *Pantoea* Plasmid 1), which is universal in all *Pantoea* spp. genomes sequenced to date, and which is thought to play a major role in the ecological diversification of *Pantoea* spp. (De Maayer et al., 2012a). A 294.6 kb contig (AHIE01000038.1) represents an incomplete copy of this plasmid in *Pnst* DC283. Thus at least ten *Pnst* DC283 contigs, with a total size of 777.6 kb, are of plasmid origin, suggesting that plasmids contribute at least 14.90% of total genomic DNA content of *Pnst* DC283, with the 845 plasmid-encoded CDSs representing 15.40% of the total genomic CDSs.

**FIGURE 4:**
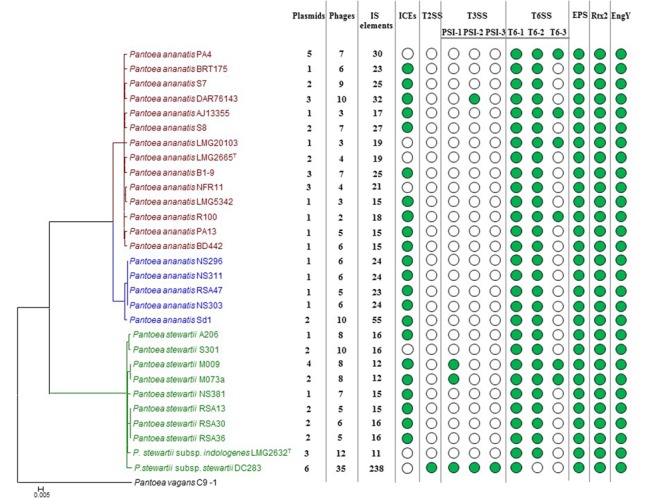
Prevalence of the mobilome and pathogenicity factors among the *Pnan* and *Pnst* strains. A core genome phylogeny is shown on the left as constructed on the basis of 2,817 core genes conserved among all compared strains. The number of predicted plasmids, prophages and insertion sequences (IS) present in each genome are indicated. Green dots indicate the presence of integrative and conjugative elements (ICEs) and pathogenicity determinants encoded on the genome.

On the basis of the genome annotations and local BlastP analyses with the *Pnst* DC283 Rep and Mob proteins, putative plasmids maintained by each of the *Pnan1, Pnan2* and *Pnst* were characterized (**Figure 4** and **Supplementary Table S2**). Three predicted plasmids are harbored in the genome of *Pnst* subsp. *indologenes* LMG 2632^T^, contributing 8.06% of the total genomic DNA content, while two plasmids are maintained by the other *Pnst* strains, with the exception of *Pnst* NS381, which only harbors a 317.5 kb LPP-1 plasmid (**Figure 4** and **Supplementary Table S2**). Aside from the universal LPP-1 plasmid, seven *Pnan1* strains harbor an additional one to four plasmids. In the case of *Pnan1* PA4, its five plasmids contribute 11.51% of the total genomic DNA content and 12.25% of the total CDSs encoded on the genome. By contrast, the genomes of the *Pnan2* strains generally only include LPP-1 plasmids, with the exception of *Pnan2* strain Sd-1 which includes an additional 104.9 kb plasmid.

Comparisons of the LPP-1 plasmids showed that a relatively high degree of synteny and conservation exist in this plasmid among the compared strains, with 148 CDSs conserved among all of the LPP-1 plasmids (42.17–61.67% of the total LPP-1 encoded CDSs). By contrast, the other plasmids are less well conserved. For example, only twenty, zero and eight out of the 529 CDSs encoded on the other *Pnst* DC283 plasmid contigs share orthology with proteins encoded on the non-LPP-1 plasmids of *Pnan1* PA4 (four plasmids; 280.4 kb; 313 CDSs), *Pnan2* Sd-1 (one plasmid; 104.9 kb; 103 CDSs) and *Pnst* LMG 2632 (two plasmids; 75.7 kb; 86 CDSs), respectively. These orthologs are largely restricted to those CDSs involved in plasmid replication, transfer and stabilization, while the plasmid cargo genes are not conserved. This would suggest that plasmids play a major role in the diversification of the *Pnan* and *Pnst* strains. Several predicted pathogenicity determinants, including a Type II secretion systems and three Type III secretion systems in *Pnst* DC283 and a Type VI secretion system in several *Pnan1* strains are encoded by these plasmid cargo genes, further suggesting an important role of plasmids in the evolution of pathogenicity mechanisms.

#### Integrative and Conjugative Elements

Previously, we have shown that Integrative and Conjugative Elements (ICEs), a class of self-transmissible integrative elements, may play an important role in the ecological diversification and antibiotic capacities of *P. ananatis* (De Maayer et al., 2015). In the current study, ICEs were present in the genomes of 22/29 compared strains, with all *Pnan2* strains carrying ICE*Pan* elements, while the genomes of 10/14 *Pnan1* and 7/10 *Pnst* strains incorporate ICE*Pan* elements (**Figure 4** and **Supplementary Table S2**). Furthermore, the genome of one strain, *Pnan1* DAR76143, incorporates two distinct ICE*Pan* elements. As was observed for the plasmids, genes coding for the excision, conjugative transfer, integration and maintenance of the ICE*Pan* elements were conserved among the different compared strains, while variability was observed in terms of the ICE*Pan* cargo genes. While ICE*Pan* element proteins only constitute a small proportion of the overall genome protein content (0.71–3.51%), the versatile nature of these elements may also contribute to the diversification of the strains carrying this element.

#### Prophages

Phages play a major role in the evolution of bacteria. Chromosomal rearrangements and deletion events associated with prophage integration and excision have been linked to strain specific differences in a range of bacterial taxa (Canchaya et al., 2003; Brüssow et al., 2004). Prophages also carry various fitness and virulence factors that contribute toward the diversification and pathogenesis of their bacterial hosts (Canchaya et al., 2003; Brüssow et al., 2004).

Prophages were detected in the genomes of all twenty-nine strains analyzed using the PHAST server (Zhou et al., 2011). Between two and ten predicted phage elements are integrated into the genomes of *Pnan1* strains (∼0.4–2 prophages integrated per Mb genomic DNA), while between six and ten prophages were identified in the *Pnan2* genomes (∼2–2.7 prophages integrated per Mb) (**Figure 4** and **Supplementary Table S2**). Similar patterns of prophage integration were observed for all of the *Pnst* strains compared, with the exception of *Pnst* DC283. In the latter strain thirty-five distinct (seven intact and twenty-eight incomplete) phage elements could be observed, with ∼7 prophages integrated per Mb of genomic DNA (**Figure 4** and **Supplementary Table S2**). These phages encode 14.84% of the total genomic proteins indicating that, along with plasmids, bacteriophage integration probably plays an important evolutionary role in this strain.

#### Insertion Sequences and Transposases

In comparison to the *Pnan1, Pnan2* and other *Pnst* genomes, a much larger proportion of the *Pnst* DC283 CDSs are involved in the “DNA replication, recombination and repair” COG function (560 CDSs – 10.20% of total genomic CDSs) (**Figure 3**). Analysis of this fraction revealed that most of the CDSs in this functional category code for up to 241 distinct transposases or their inactivated derivatives. By contrast, analysis of the annotations of the other genomes revealed that only between eleven (*Pnst* LMG 2632) and fifty-three (*Pnan2* Sd-1) transposases are encoded on the genomes (**Supplementary Table S2**). Insertion Sequences (ISs) were also predicted from the genome sequences using the ISSaga server (**Figure 4** and **Supplementary Table S2**) (Varani et al., 2011). Among the *Pnan1* strains, between 15 and 32 ISs, belonging to between three and nine IS families, constitute an average 1.45% of the total genomic DNA. Between 23 and 55 ISs (1.28–3.16% of the total genomic DNA), belonging to between three and nine IS families, are incorporated in the *Pnan2* genomes. By contrast, with the exception of *Pnst* DC283, far fewer ISs are integrated into the genomes of the other *Pnst* strains (between 11 and 14 ISs; 0.63–1.07% of the total genomic DNA). A total of 238 distinct IS elements were predicted in the genome of *Pnst* DC283, with a total predicted size of 802.8 kb, constituting 15.38% of the total genomic DNA content (**Figure 4** and **Supplementary Table S2**). The ISs incorporated in the genome of this strain are also more diverse, belonging to sixteen distinct families, with the majority belonging to the IS66 (101) and IS630 (64) families.

### The *Pnan* and *Pnst* Strains Share Several Common Pathogenicity Determinants but Vary in Terms of Their Secretion Systems

**FIGURE 5:**
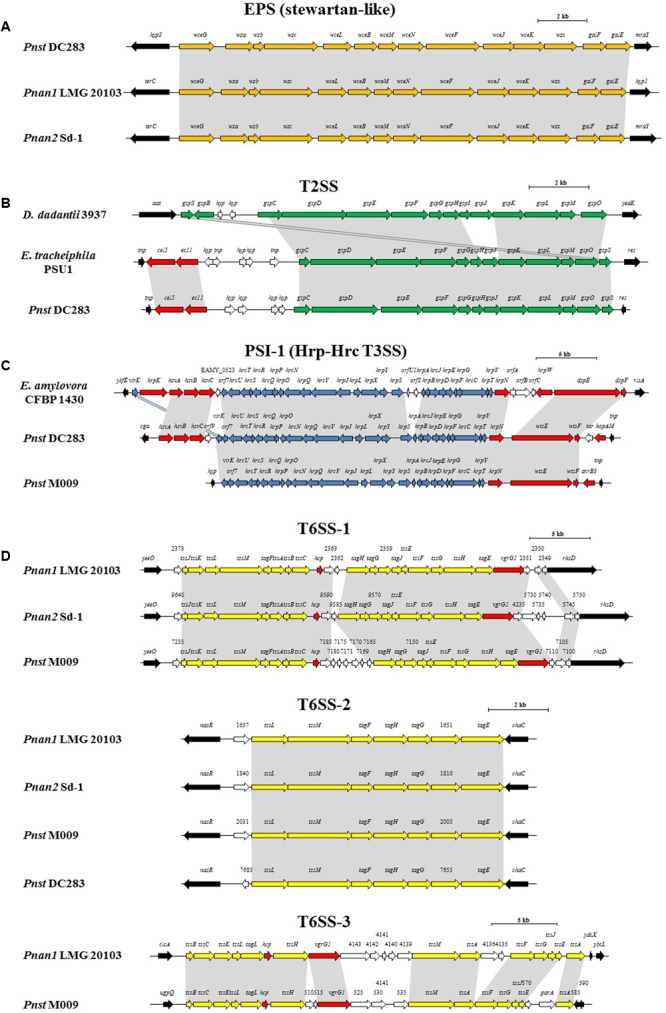
Alignments of the loci encoding for the predicted pathogenicity determinants from representative strains of each clade, *Pnan1, Pnan2* and *Pnst* as well as representatives of other genera. **(A)** stewartan-like EPS (orange), **(B)** T2SS (green), **(C)** Hrp-Hrc T3SS (blue) and **(D)** T6SS-1, T6SS-2 and T6SS-3 loci (yellow). Red arrows indicate genes coding for predicted effectors of the T3SS and T6SSs, white arrows indicate genes coding for hypothetical proteins and black arrows denote the flanking genes. Size bars indicate the estimated sizes of the loci.

While little is known about the pathogenicity determinants of *P. ananatis*, the pathogenicity and virulence factors of the maize pathogenic *Pnst* subsp. *stewartii* have been extensively studied (Roper, 2011). During the early infection phase, *Pnst* subsp. *stewartii* is introduced into the plant apoplast by feeding corn flea beetles, where it subsequently causes cellular damage and cell content leakage, resulting in water soaked lesions. A recent study has attributed this activity to a large repetitive RTX-like toxin, Rtx2 (Roper et al., 2015). Analysis of the other genomes showed that this protein is well conserved among all *Pnan* and *Pnst* genomes (74.96% average amino acid identity; *E*-value: 0.0) (**Figure 4**), and hence the water-soaked lesion symptoms observed in maize, rice and *Eucalyptus* infected with *P. ananatis* (Coutinho et al., 2002; Bomfeti et al., 2008; Mondal et al., 2011) may be attributed to this pathogenicity factor. During the subsequent system infection phase of Stewart’s vascular wilt, *Pnst* subsp. *stewartii* colonizes the xylem vessels, where it produces an exopolysaccharide, stewartan that results in vascular occlusions and vascular wilt (Wang et al., 2012). Stewartan biosynthesis is dependent on a single ∼18.4 kb genetic locus (AF077292.2) which codes for fourteen proteins. Highly conserved homologous and syntenous loci (92.30% average nucleotide identity) are present in all the *Pnst* and *Pnan* genomes compared (**Figures 4, 5A**) and may encode exopolysaccharides with similar structures and functions as stewartan. Additionally, *Pnst* DC283 produces an endoglucanase, EngY (CKS_4400), which has both glucanolytic and xylanolytic activities and has been shown to play a role in the systemic spread of this pathogen within its maize host (Mohammadi et al., 2012). Orthologs (94.39% average amino acid identity; *E*-value: 0.0) of this enzyme are encoded on the genomes of all the *Pnan* and *Pnst* strains compared (**Figure 4**).

The presence of highly conserved orthologs of these *Pnst* subsp. *stewartii* pathogenicity determinants in all the *Pnan* and *Pnst* strains compared suggests they use common mechanisms to effect pathogenesis and symptom development. However, substantial differences can be observed in terms of the secretion systems encoded on their genomes, which have been shown to play an important role in the phytopathogenesis of both species. Analysis of the *Pnst* DC283 genome shows the presence of a locus coding for a Type II secretion system (T2SS; CKS4616-CKS4630) on the pSW1200 plasmid contig (AHIE01000039.1), unique among the strains compared (**Figures 4, 5B**). While the *Pnst* DC283 T2SS remains uncharacterized, in the related phytopathogen *Dickeya dadantii* it secretes a range of plant cell wall degrading enzymes, including endo-pectate lyases, esterases, a rhamnogalacturonate lyase and a cellulase (Hugouvieux-Cotte-Pattat et al., 2014; Pineau et al., 2014). The thirteen proteins encoded by the *Pnst* DC283 T2SS locus share highest orthology with the T2SS proteins of *Erwinia tracheiphila* PSU-1 (ETR_18611–18666/22561–22566; 85.51% average amino acid identity). Adjacent to the *Pnst* DC283 locus are two proteins which are likely to represent secreted effector proteins, including a predicted plant cell wall degrading cellulase (Cel5 – CKS4630; 63% amino acid identity to *D. dadantii* 3937 P07103.2; *E*-value: 1*e*-127) and an expansin-like protein (Exl1 – CKS4629; 59% amino acid identity to *Pectobacterium carotovorum* AHH91628.1; *E*-value: 5*e*-82) which is predicted to loosen the plant cell wall and allow the pathogen to gain access to the loosened cellulose (Chapon et al., 2001; Olarte-Lozano et al., 2014).

Type III secretion systems (T3SSs) are complex extracellular injection apparatuses that deliver effector proteins into plant and animal cells, where they play a role in colonization, modulation of host defenses and pathogenesis (Correa et al., 2012; Kirzinger et al., 2015). Three different T3SSs have been shown to be encoded in three distinct *Pantoea* secretion islands (PSI-1 to PSI-3) on the genome of *Pnst* DC283. The PSI-1 (Hrc-Hrp 1) T3SS (CKS_3241–3281), encoded on the LPP-1 plasmid contig (AHIE01000038.1), is similar to that found in other enterobacterial plant pathogens including *Erwinia, Dickeya* and *Pectobacterium* spp. and is central to *Pnst* DC283 pathogenesis on maize (Correa et al., 2012; Kirzinger et al., 2015). Analysis of the compared genomes showed that the PSI-1 T3SS is restricted to only four *Pnst* strains, *Pnst* M009, M073a, LMG 2632 and DC283 (**Figure 4**). While the PSI-1 T3SS loci are highly conserved (29 conserved CDSs; 99.54% average amino acid identity) and syntenous in terms of their structural and functional gene complements, differences could be observed in terms of the predicted effectors encoded within these loci (**Figure 5C**). In particular, among the PSI-1 encoding *Pnst* strains, only the *Pnst* DC283 Hrp T3SS incorporates *hsvA, hsvB* and *hsvC* genes at its 5′ end. Orthologs of the encoded proteins in *E. amylovora* (AAX39432-4.1; 72.25% average amino acid identity) are required for systemic virulence in apple and may be involved in the synthesis of a phytotoxin (Oh et al., 2005). The *Pnst* DC283 PSI-2 (CKS_4512–4544), encoded on the pSW1200 plasmid (AHIE0100039.1), is orthologous to a T3SS found in animal pathogens such as *Salmonella enterica* and *Yersinia enterocolitica* and plays a role in *Pnst* DC283 persistence in its corn flea beetle vector (Correa et al., 2012). Among the strains compared, PSI-2 T3SSs are restricted to *Pnst* DC283 and *Pnan1* DAR76143 (BATH01000023.1; 25 proteins; 64.66% average amino acid identity) (**Figure 4**). The third T3SS, PSI-3, is likewise encoded on pSW1200 (AHIE01000039.1) and is evolutionarily related to a T3SS in *Salmonella* spp., but its function in *Pnst* DC283 remains unknown (Kirzinger et al., 2015). No orthologous PSI-3 T3SS loci are encoded on the genomes of the other twenty-eight *Pnan* and *Pnst* genomes (**Figure 4**).

Type VI secretion systems (T6SSs) are macromolecular injectisomes that are widely distributed among Gram-negative bacteria and have been implicated in a wide range of functions, including biofilm formation, plant and animal host cell invasion and virulence, as well as in bacterial competition (Coulthurst, 2013; Cianfanelli et al., 2016). Previously, we have shown that up to three distinct T6SSs (T6SS-1 to T6SS-3) are encoded on the genomes of different *Pnan* strains (**Figures 4, 5D**) (De Maayer et al., 2011, 2014). T6SS-1 is conserved among all *Pnan1, Pnan2* and *Pnst* strains, with the exception of *Pnst* DC283 and has been implicated in both bacterial competition and virulence on onion seedlings in *Pnan1* LMG 2665^T^ (Shyntum et al., 2015). The T6SS-2 locus is conserved among all twenty-nine compared strains, including *Pnst* DC283 (**Figure 4**). This locus encodes only seven of the core proteins required for T6SS biosynthesis, and knock-out deletion does not have any effect on the virulence and antibiosis phenotypes (Shyntum et al., 2015). As such, it has been postulated that this locus encodes a non-functional T6SS and was derived through partial duplication of the T6SS-1 locus (De Maayer et al., 2011). In contrast to the other two loci, the T6SS-3 locus shows a more restricted distribution (**Figure 4**), being found on the LPP-1 plasmid of only four *Pnan1* strains (AJ13355, LMG 20103, PA4 and R100). No orthologous loci could be observed in the genomes of any of the *Pnan2* strains, but is present in two *Pnst* strains (M009 and M073a) where it is predicted to reside on the chromosome. Alignments of the T6SS loci shows that these loci are highly conserved and syntenous (**Figure 5D**). Non-conserved islands of genes are, however, present in the T6SS-1 and T6SS-3 loci adjacent to the *hcp* and *vgrG* genes, which code for predicted effector proteins of the T6SS (Durand et al., 2014). It is postulated that these non-conserved genes represent effector domains, which are tagged onto the Hcp and VgrG effector proteins and subsequently delivered into the environment or host cell (De Maayer et al., 2011; Durand et al., 2014). Orthology searches of the protein products of these non-conserved genes from eight *P. ananatis* strains showed that the T6SS-1 genes are restricted in distribution to plant-associated bacteria, while those from T6SS-3 are restricted to animal-associated bacteria, suggesting that these two T6SSs may be involved in plant and animal host interactions, respectively (De Maayer et al., 2014).

## Discussion

*Pnan* strains, and some *Pnst* strains, are readily isolated from a wide range of environmental sources and cause disease on a broad range of plant hosts. By contrast, *Pnst* subsp. *stewartii* strains have a restricted lifecycle in that they rely on specific insect hosts for survival and transmission and have a narrow host range, causing disease only in susceptible maize varieties. The availability of a large number of genome sequences and a broad range of *in silico* tools has provided an enticing opportunity to determine the molecular differences underlying their distinct lifestyles.

Phylogenomic analyses indicated that there is extensive phylogenetic diversity among the *Pnan* strains, and that these strains form two distinct clades. *Pnan* Clade 1 incorporates the original type strain of the species, *Pnan* LMG 2665^T^ and includes seven phytopathogenic strains (of maize, rice, pineapple, onion and *Eucalyptus*), three endophytic strains (from rice, maize and switchgrass), one epiphytic strain (on strawberry), one soil isolate, one plant-growth promoting strain and a clinical isolate. *Pnan* Clade 2 incorporates five strains, all of which were isolated as endophytes from rice seeds. Overall, less variability was observed in terms of the proteins encoded on the *Pnan2* genomes than the *Pnan1* strains, which may be correlated to the restricted source of isolation and clonality of most of the *Pnan2* strains. As many of the genome sequences were deposited without information on its origin and ecology, additional analyses will need to be undertaken to identify cohesive and defining phenotypic differences between the *Pnan1* and *Pnan2* strains (Brenner et al., 2006). Far less phylogenetic diversity was observed among the *Pnst* strains, when considering the core genome phylogeny and phylogenomic metrics.

We propose that the different lifestyle of the *Pnan* and *Pnst* strains may largely be attributed to the distinct mobilomes observed among the compared strains. The *Pnan1* strains harbor between one and five plasmids, the *Pnan2* strains one or two plasmids, while the *Pnst* strains harbor between one and six distinct plasmids. These plasmids contribute heavily toward the total proteins encoded on the genomes (up to 15.40% in *Pnst* DC283) and form a substantial part of the accessory and variable fraction of the genomes. Plasmid-borne features with a more restricted distribution include the T2SS and the T3SSs, with predicted roles in pathogenicity and virulence. *Pnst* DC283 is also particularly prone to prophage integration, with 35 predicted prophages integrated on its chromosome, while between two and twelve phages are integrated into the genomes of the other *Pnan1, Pnan2* and *Pnst* strains. Together, plasmids and prophages are predicted to play a major role in shaping the genomes of these distinct species and strains, contributing between 7.61 (*Pnan1* R100) and 28.18% (*Pnst* DC283) of the total genomic DNA content and comprising largely of those proteins which form part of the accessory genome fractions of the compared strains.

Another prominent feature of the *Pnst* DC283 genome is the large number of insertion sequences (238 distinct IS elements) and transposons (240 distinct transposase proteins) integrated into the genome. While the direct phenotypic effects of transposon and insertion sequence integration in *Pnst* DC283 cannot be determined without further laboratory analyses, their integration may have contributed toward the non-deleterious “knock-out” of those phenotypes not required within the limited environmental context in which it survives (Shapiro et al., 2016), thereby restricting the metabolic and energy costs associated with these phenotypes and the genomic replication of the DNA coding for these phenotypes. Reductive genome evolution or pathoadaptation has been described in the xylem-limited plant pathogens *Xylella fastidiosa* and *Xanthomonas albilineans* and a number of obligate animal symbionts and pathogens, where initial accumulation of mobile genetic elements results in pseudogenization (gene inactivation) and ultimate gene loss (Pieretti et al., 2009; Lindeberg, 2012). Similar loss of epiphytic fitness factors and reduction in genome size, combined with acquisition of Hrp T3SS (PSI-1) and exopolysaccharide biosynthesis genes has been observed in the more closely related phytopathogens *Erwinia amylovora* and *Erwinia tracheiphila* (Kamber et al., 2012; Shapiro et al., 2016). The presence of large numbers of mobile elements in the genome of *Pnst* DC283 suggests that pathoadaptation into a host-specific pathogen with a restricted lifecycle is the result of recent evolutionary events or is still on-going (Shapiro et al., 2016). Genome sequencing and analyses of other Stewart’s wilt *Pnst* subsp. *stewartii* isolates may provide better insights into the distinct evolutionary history of this subspecies.

While the ten *Pnst* strains form a phylogenetically distinct cluster from the *Pnan1* and *Pnan2* strains, the nine *Pnst* strains (except *Pnst* DC283) share several genomic features with the *Pnan* strains that differentiate them from *Pnst* DC283. When comparing *Pnan1*+ *Pnan2*+ *Pnst*, the core genomes were consistently larger (on average 371 CDSs more) when the non-DC283 *Pnst* strains were considered. A broader profile of metabolic proteins was also encoded on their core and accessory genome fractions compared to *Pnst* DC283. This suggests that the *Pnan1, Pnan2* and other *Pnst* strains are comparatively better adapted for survival in various environmental niches. Furthermore, as they will have to effectively outcompete other bacteria for available nutrients in these various environmental niches, they may make use of the type VI secretion systems (De Maayer et al., 2011) and the putative antimicrobial encoded on the ICE*Pan* elements (De Maayer et al., 2015) integrated into the genomes of most of the *Pnan* and *Pnst* strains compared. A pertinent finding from the genomic analysis was that although they differ distinctly in terms of their host range and lifestyle, many predicted pathogenicity determinants are conserved among all compared *Pnan* and *Pnst* strains, regardless of whether they were isolated from diseased plant material, as part of the endophytic or epiphytic flora or from an environmental source. These putative pathogenicity determinants include an Rtx2 toxin which has been linked to the development of water-soaked lesions on the plant host (Roper et al., 2015), a stewartan-like exopolysaccharide (Wang et al., 2012), and the glucanolytic/xylanolytic endoglucanase EngY (Mohammadi et al., 2012). This shared pathogenicity factor profile, along with the extensive core genome observed among the twenty-nine compared strains emphasize the common ancestry of *Pnan* and *Pnst*, and further highlights the complex evolutionary histories and diversification of these important phytopathogens.

## Availability of Data and Materials

All genome sequences incorporated in this study are publically available in the NCBI Genome database or the Joint Genome Institute – Integrated Microbial Genome Database (JGI-IMG). The accession numbers for the genome sequences are indicated in **Table 1**.

## Author Contributions

PDM, BD, TS, SNV, and TC conceived the study. PDM, HA, SV, and JB performed data analyses. PDM wrote the original manuscript and all authors contributed to the final version. All authors have read and approved the final manuscript.

## Conflict of Interest Statement

The authors declare that the research was conducted in the absence of any commercial or financial relationships that could be construed as a potential conflict of interest.

## Footnotes

1 http://www.bacterio.net/pantoea.html

2 https://www.ncbi.nlm.nih.gov/genome

3 https://ggdc.dsmz.de

## Supplementary Material

TABLE S1 Digital DNA-DNA hybridization (dDDH) values and Average Nucleotide Identity (ANI) values between the twenty-nine compared *Pnan1, Pnan2* and *Pnst* strains. ANI values are in the top half, while dDDH values are in the bottom half of the table. Values from 70 to 100% are colored in a sliding scale from light gray to black.

TABLE S2 Mobile genetic elements among the twenty-nine compared *Pnan* and *Pnst* strains. The number of plasmids, phages, ICEs, IS elements and transposases are tabulated and their contribution to the total genomic DNA (%) and total proteins (%) encoded on the genome are described.
